# Application of the hollow fibre infection model (HFIM) in antimicrobial development: a systematic review and recommendations of reporting

**DOI:** 10.1093/jac/dkab160

**Published:** 2021-06-28

**Authors:** Zahra Sadouki, Timothy D. McHugh, Rob Aarnoutse, Julio Ortiz Canseco, Christopher Darlow, William Hope, Jakko van Ingen, Christopher Longshaw, Davide Manissero, Andrew Mead, Ludovic Pelligand, Lynette Phee, John Readman, Mike M. Ruth, Joseph F. Standing, Neil Stone, Emmanuel Q. Wey, Frank Kloprogge

**Affiliations:** 1 Institute for Global Health, University College London, London, UK; 2 Centre of Clinical Microbiology, University College London, London, UK; 3 Department of Internal Medicine, Radboud Center of Infectious Diseases (RCI), Radboud University Medical Center, Nijmegen, The Netherlands; 4 Antimicrobial Pharmacodynamics and Therapeutics, University of Liverpool, Liverpool, UK; 5 Department of Medical Microbiology, Radboud University Medical Center, Nijmegen, The Netherlands; 6 Medical Affairs, Shionogi Europe, London, UK; 7 Medical Affairs for Infection and Immune Diagnostics, QIAGEN, London, UK; 8 Department of Comparative Biological Sciences, The Royal Veterinary College, London, UK; 9 Antimicrobial Research Group, Barts and The London School of Medicine and Dentistry, Queen Mary University London, London, UK; 10 Infection, Immunity, Inflammation Section, University College London Great Ormond Street Institute of Child Health, London, UK; 11 Department of Microbiology, University College London Hospitals, London, UK; 12 Royal Free London NHS Trust, London, UK

## Abstract

**Objectives:**

This systematic review focuses on the use of the *in vitro* hollow fibre infection model (HFIM) for microbial culture. We summarize the direction of the field to date and propose best-practice principles for reporting of the applications.

**Methods:**

Searches in six databases (MEDLINE^®^, EMBASE^®^, PubMed^®^, BIOSIS^®^, SCOPUS^®^ and Cochrane^®^) up to January 2020 identified 129 studies meeting our inclusion criteria. Two reviewers independently assessed and extracted data from each publication. The quality of reporting of microbiological and technical parameters was analysed.

**Results:**

Forty-seven out of 129 (36.4%) studies did not report the minimum pharmacokinetic parameters required in order to replicate the pharmacokinetic profile of HFIM experiments. Fifty-three out of 129 (41.1%) publications did not report the medium used in the HFIM. The overwhelming majority of publications did not perform any technical repeats [107/129 (82.9%)] or biological repeats [97/129 (75.2%)].

**Conclusions:**

This review demonstrates that most publications provide insufficient data to allow for results to be evaluated, thus impairing the reproducibility of HFIM experiments. Therefore, there is a clear need for the development of laboratory standardization and improved reporting of HFIM experiments.

## Introduction

**Figure 1.**
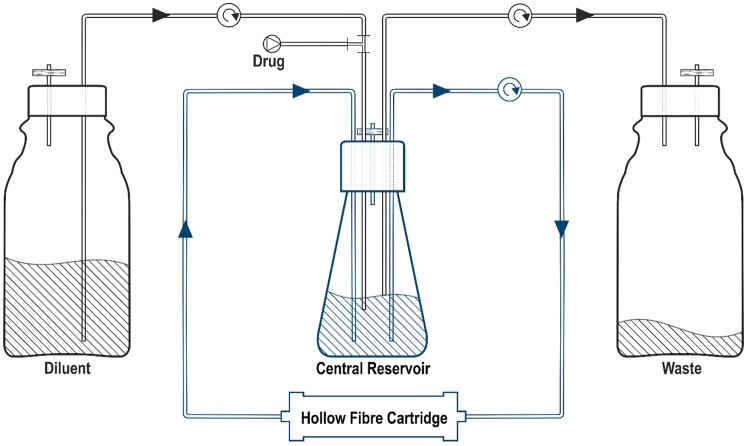
Schematic of the HFIM. This schematic shows the hollow fibre compartment model. The hollow fibres in the cartridge are attached to a circuit connecting to a central reservoir (shown in blue). Test organisms are retained in the hollow fibre cartridge. The contents of the central reservoir can be topped up with fresh medium from the diluent compartment (shown on the left) and through the use of a waste removal tube the volume of the central reservoir is kept constant. Drug is administered directly into the central reservoir through the diluent tubing. This figure appears in colour in the online version of *JAC* and in black and white in the print version of *JAC*.

The hollow fibre infection model (HFIM) is an *in vitro* system that offers a solution to culturing cells continuously at high density with flexibility and reproducibility.^1^ Applications range from the propagation of cell lines and the production of monoclonal antibodies and recombinant proteins to mimicking long-term physiologically relevant *in vivo* profiles.^2–5^ The HFIM is a preclinical closed system that allows the culturing of microbial cultures in an enclosed compartment. This compartment is usually a discrete cartridge that in turn is threaded with semi-permeable fibres.^6^^,^^7^ These fibres are attached to a circuit connecting to a central reservoir where the contents are rapidly circulated via a pump and nutrients and drugs equilibrate freely between the central reservoir circuit and the inoculum-containing compartment of the cartridge (Figure 1). Fresh medium is supplemented into the central compartment at a fixed rate, with central compartment contents removed via a pump at an identical rate. Through this, the drug in the central compartment is cleared at a rate determined by the supplementation rate. Adjustment of input/output rates allows simulation of clearance of the drug(s) added to the central reservoir, mimicking pharmacokinetic (PK) profiles seen *in vivo*. Samples can be taken from the enclosed compartment of the hollow fibre cartridge for quantification of the bacterial inoculum and determination of antimicrobial resistance. Samples can be taken from the central compartment to quantify the concentration of drug via bioanalysis to confirm recapitulation of the mimicked PK profile.

Over recent years, the HFIM has been increasingly used to characterize *in vitro* pharmacodynamics (PD) of antimicrobial agents and to determine PK-PD indices, such as AUC/MIC, *T*_>MIC_ and *C*_max_/MIC.^8^^,^^9^ The model allows physiological PK drug profiles to be simulated with serial evaluation of the interplay between drug exposure and microbial response. In contrast, conventional *in vitro* infection models have generally relied on static time–kill experiments or dynamic one-compartment models with the microbial load in the central compartment.^10^^,^^11^ These latter models mimic drug concentrations by supplementation and removal of medium, such as in the HFIM, but cause continual dilution of the bacterial population. Furthermore, drug concentrations may be inaccurate due to difficulties of bioanalysis in infected medium and consumption of the drug by the microbial load.^12^^,^^13^ In contrast, in an HFIM, the microbial cell cultures remain contained in a separated compartment, whilst mimicking physiological PK profiles, allowing for accurate determination of drug concentration and no continual dilution of bacteria.^14^^,^^15^

The HFIM also has advantages over *in vivo* animal models. These *in vivo* models, particularly the thigh infection model, are well established and frequently used as preclinical models for anti-infective drug development.^16–18^ The advantages and disadvantages of both methods are well documented in publications; here we highlight the main advantages of the HFIM. Importantly, the life of an animal limits the duration of animal experiments and multiple animals need to be offered in each arm for each timepoint.^19^ Therefore, animal experiments are rarely long enough to quantify the emergence of resistance; however, the HFIM allows for higher sampling frequency over longer time periods, which enables the understanding of the PD of the emergence of resistance.^20–23^  *In vivo* animal models are also limited to bacterial loads that the animals can maintain over a period of time. In contrast the HFIM allows for high bacterial inocula to be maintained without issue. Furthermore, *in vivo* animal models rely on humanized doses, whilst needing to account for differences in elimination and distribution characteristics, such as metabolism and protein binding, whereas any PK parameter can be quite precisely mimicked in the HFIM.^24^ Animal models raise ethical concerns and require ethical approval of the experimental protocol, whereas culturing microbes in the HFIM does not. Ultimately, the HFIM provides more flexibility in experimental design and sampling.

Whilst there are established CLSI guidelines for static time–kill experiments, no comprehensive recommendations or standards (CLSI or otherwise) for performance of hollow fibre microbial experiments exist.^25^^,^^26^ Laboratory manuals are available, but consensus guidelines are currently lacking.^27^^,^^28^ Furthermore, the current literature lacks a systematic review detailing the experimental details of HFIM applications reported in original research papers. This, along with the high running costs and infrastructure requirements, forms a barrier to entry for groups new to the field, thus limiting the evaluation and reproducibility of published results. The aim of this work was therefore to undertake a systematic review of HFIM publications, describing the current state of the art with the objective of reviewing the reporting of data.

## Methods

We included publications that reported use of the HFIM, describing the experimental aims as well as the methodology and outcomes. This review represents the direction of the field of HFIM research to date and focuses on *in vitro* infection models for microbial cell culture with the following objectives: (i) to describe the primary aims of the use of hollow fibre systems; (ii) to evaluate the experimental settings and parameter reporting; and (iii) to evaluate the microbiological outcome measure reporting.

### Search strategy

PRISMA 2020 guidelines^29^ were followed and the following databases were searched for relevant records: MEDLINE^®^, EMBASE^®^, PubMed^®^, BIOSIS^®^, SCOPUS^®^ and Cochrane^®^. The search strategy included three concepts: microbes, antimicrobials and the hollow fibre system. A detailed breakdown of the PICO framework alongside search concepts can be found in Table S1 (available as Supplementary data at *JAC* Online). All six databases were searched with our predefined search terms and our search strategy for each database is captured in Table S2. We included records published in English from January 1980 to January 2020.

### Inclusion and exclusion criteria

**Figure 2.**
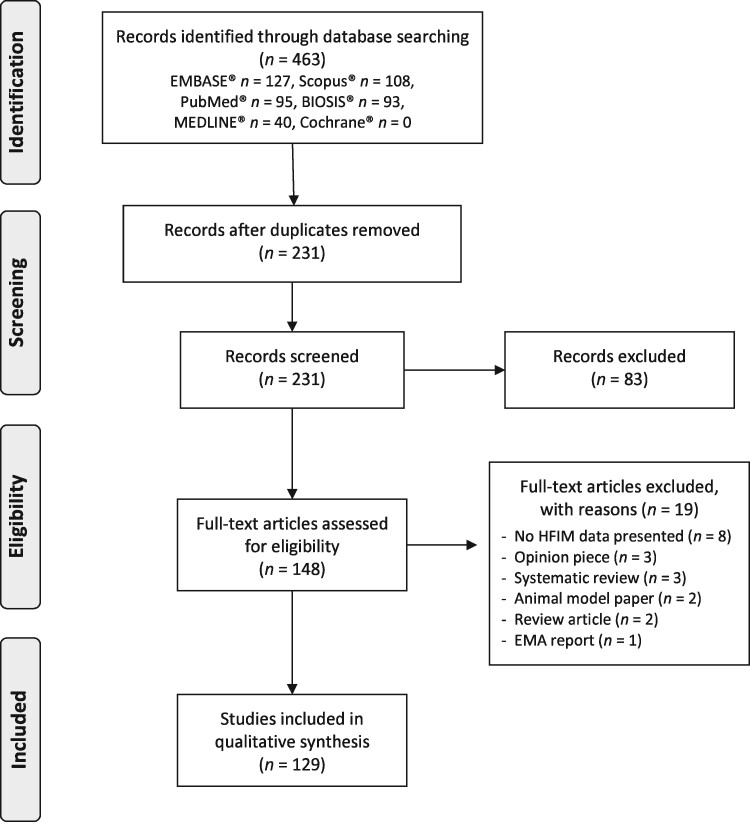
PRISMA flowchart outlining the flow of information through the different phases of the systematic review, including the number of records identified, included and excluded.

Search results were de-duplicated in the referencing software Mendeley© and all unique records were screened for the relevance of their title and abstract. We defined the inclusion criteria as records that: (i) presented primary hollow fibre data; (ii) studied microorganisms; and (iii) studied antimicrobials. Publications were excluded at the screening stage if their title and abstract did not meet the inclusion criteria. This included studies that did not use the HFIM, did not investigate microbial species and did not investigate antimicrobials. Screened abstracts were then assessed for their full-text eligibility by two independent reviewers in the data extraction phase. At full-text screening further papers were excluded if they didn’t fulfil the inclusion/exclusion criteria. All records excluded from the review at full-text screening are presented in the PRISMA flowchart diagram with details of their exclusion (Figure 2).

### Data extraction

Data extraction was performed on full-text articles in duplicate by two independent authors, Z.S. extracted data from all publications and the duplicate extractions were shared amongst all collaborating authors. A password-protected Rshiny form was designed using shiny version 1.4.0 to enable robust data extraction.^30^ The form was separated into three sections: (i) descriptive analysis of the records; (ii) engineering parameters; and (iii) the microbiological parameters. We defined the engineering parameters as settings or equipment that are not directly related to the microbiological measurements, but which impact the experimental dynamics. These included settings that determine the PK profile to be mimicked (e.g. pump settings and PK parameters), cartridge/fibre type and the tubing length and bore size. The microbiological section included parameters that influence the growth of the microorganisms or measures that arise from the sampling of the microorganism to determine the PD response to the antimicrobial. Technical repeats were defined as repeat testing of endpoints, for example bacterial quantification or MIC. Biological repeats were defined as repeat experiments of the organism investigated. Details of the parameters extracted alongside the definitions we used can be viewed in Table S3.

## Results

The results are separated into four sections: (i) PRISMA flowchart; (ii) a descriptive analysis of records included; (iii) engineering parameters; and (iv) the microbiological parameters.

### Section one: PRISMA flowchart

A PRISMA flowchart (Figure 2) outlines the number of publications at each stage of the systematic review.

Following deduplication, a total of 231 independent publications for title and abstract screening remained. Of these 83 were excluded and 148 were eligible for full-text screening. Upon full-text screening, 19 records were excluded and a total of 129 records were included in the data extraction and analysis.

### Section two: Description of records included

The three most common aims of the HFIM experiments were investigating drug combinations [38.8% (50/129)], investigating antimicrobial resistance [26.4% (34/129)] and determining PK-PD indices [21.7% (28/129)] (Table 1). A few publications modelled intravascular time course [3.9% (5/129)] and the remaining 9.3% (12/129) ranged from investigating intracellular pathogens to investigating PK assay development. Further information and breakdown of the microbial species and antimicrobials investigated in the publications included is provided in Table S4.

**Table 1.** HFIM publication aims frequency

Primary publication aims	Number	Percentage
Drug combinations	50	38.8
Antimicrobial resistance	34	26.4
Determining PK-PD indices (drug development)	28	21.7
Modelling intravascular time course	5	3.9
Intracellular pathogens	4	3.1
Determine conditions for drug production	2	1.6
Dose finding	2	1.6
Other	4	3.1
Total	127	100

### Section three: Technical specifications

Simulating a PK profile is achieved by adjusting the HFIM settings, namely the pump settings. Technical settings play a vital role for interpreting the results and enabling reproducibility. *C*_max_, *T*_max_ and *t*_½_ are required in order to replicate a PK profile of an extravascularly administered drug or intravenously administered drug by infusion (in which case *T*_max_ is usually the infusion duration). For intravenous bolus administration only *C*_max_ and *t*_½_ are required.

**Figure 3.**
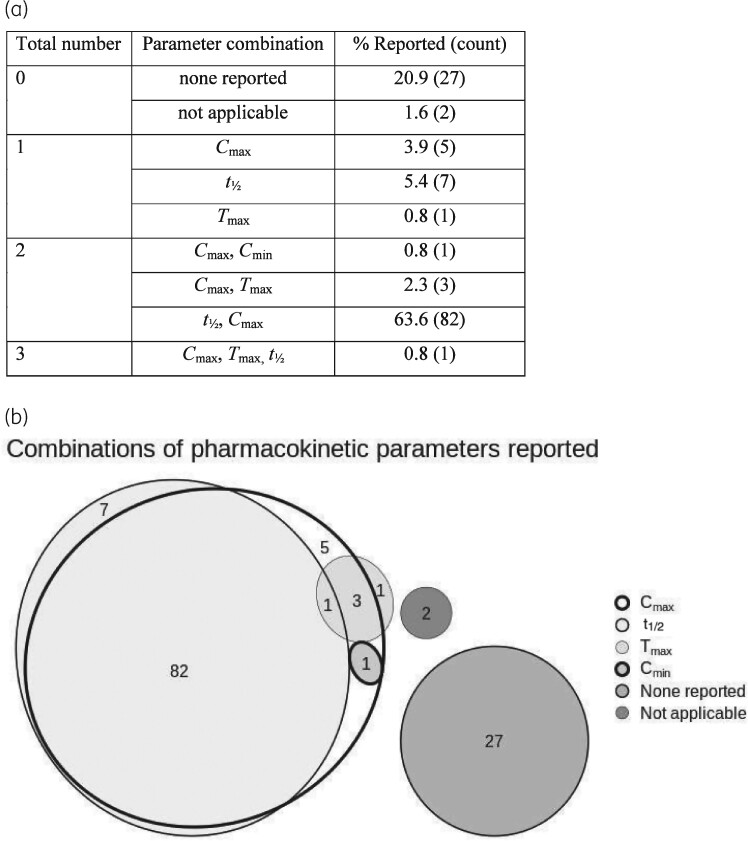
Summary of PK parameter reporting in HFIM studies, presented in table format (a) and as a Euler proportional diagram (b).

Twenty-seven out of 129 (20.9%) publications did not report any PK parameters. *C*_max_ and *t*_½_ were the most reported PK parameters with 37.0% and 36.6% of total publications reporting them, respectively. Antimicrobial dose mimicked was reported by 45.7% of publications. Eighty-two out of 129 (63.6%) reported both *C*_max_ and *t*_½_ - the minimum required to replicate the PK profile of intravenous bolus administration. Only one publication reported all of *C*_max_, *T*_max_ and *t*_½_, the minimum required to replicate the PK profile of drugs administered extravascularly, or intravascularly via infusion. In summary, 47/129 (36.4%) publications did not meet the minimum criteria for reproducibility of PK profiles (Figure 3).

Seventy-two out of 129 (55.8%) publications reported the manufacturer of the cartridge used in the hollow fibre experiments and 37/129 (28.7%) publications reported the type of fibre used by either reporting the cartridge catalogue number or explicitly mentioning the fibre (e.g. cellulosic, polysulfone or polypropylene). Only 6/129 (4.7%) publications reported monitoring of pH in the system. Bore size of the tubing used was reported by 3/129 (2.3%) publications. No publications reported the length of the tubing used in any part of the HFIM system (Table 2).

**Table 2.** Percentage and raw number of publications reporting hollow fibre settings

Hollow fibre setting	% Reported (*n*)	% Not reported (*n*)	% Not applicable (*n*)
Cartridge source	55.8 (72)	44.2 (57)	0.0 (0)
Fibre type	28.7 (37)	71.3 (92)	0.0 (0)
Pump dynamics	6.2 (8)	93.0 (120)	0.8 (1)
pH	4.7 (6)	94.6 (122)	0.8 (1)
Tubing bore size	2.3 (3)	96.9 (125)	0.8 (1)
Tubing length	0 (0)	99.2 (128)	0.8 (1)
Dose administration	74.4 (96)	24.8 (32)	0.8 (1)
Dose mimicked	45.7 (59)	54.3 (70)	0 (0)

### Section four: Microbiology

One hundred and eighteen out of 129 (91.5%) publications reported the results for a control experiment—often an antimicrobial-free growth control. Seventy-five out of 129 (58.2%) publications reported the medium used in the HFIM. None of the 129 publications reported sampling and monitoring for contamination. One hundred and eight out of 129 publications (83.7%) quantified the bacterial population by cfu to determine the antimicrobial killing effect. Only 6/129 (4.7%) measured markers of cell viability beyond growth (e.g. using flow cytometry). Antimicrobial resistance was measured and reported by 95/129 (73.6%) publications; however, only 27/129 (20.9%) publications reported genotypic analysis of samples taken from the hollow fibre cartridge (Table 3).

**Table 3.** Percentage and raw number reporting of microbiological outcome measures

Outcome measure	% Reported (*n*)	% Not reported (*n*)	% Not applicable (*n*)
Medium	58.14 (75)	41.09 (53)	0.78 (1)
Contamination	0.0 (0)	99.22 (128)	0.78 (1)
Control	91.47 (118)	5.43 (7)	3.10 (4)
Inoculum	76.74 (99)	20.16 (26)	3.10 (4)
cfu	83.72 (108)	13.95 (18)	2.33 (3)
Viability	4.65 (6)	91.47 (118)	3.88 (5)
Resistance	73.64 (95)	23.26 (30)	3.10 (4)
Genotyping	20.93 (27)	75.97 (98)	3.10 (4)

The overwhelming majority of publications did not perform any technical repeats [107/129 (82.9%)] or biological repeats [97/129 (75.2%)] (Table 4). Duplicate testing (i.e. one original test and one repeat test) were performed by 18/129 (14.0%) publications for technical repeats and by 23/129 (17.8%) publications for biological repeats. Four publications (3.1%) reported technical repeats in triplicate and nine publications (7.0%) reported biological repeats in triplicate.

**Table 4.** Percentage and raw number of publications reporting repeat testing in HFIM

Number of repeats	% Technical repeats (*n*)	% Biological repeats (*n*)
Single	82.9 (107)	75.2 (97)
Duplicate	14.0 (18)	17.8 (23)
≥Triplicate	3.1 (4)	7.0 (9)

## Discussion

To the best of our knowledge, this is the first systematic review focusing on the reproducibility of the microbial applications of the HFIM. In this systematic review of *in vitro* hollow fibre PK-PD studies of antimicrobials, we found wide variability in reporting. Most studies did not provide enough information for their results to allow comparison, reproduction or modification of HFIM studies. This review demonstrates that the reproducibility of published HFIM work remains impaired by the practice of inadequate reporting of the hollow fibre settings. We found many publications outsourced their methodology to previously published papers.^31–34^ This practice leaves too much interpretation and creates a further barrier for the sourcing of important information, as the specifics are often under several layers of prior publications with the referenced publications often in a different context. We would recommend that study design and methodology is restated in each individual paper to provide clarity. A systematic review of clinical PK-PD methodologies reported similar findings in lack of conduct and reporting, further highlighting the need for the field to standardize reporting.^35^

Future HFIM studies should be designed and reported carefully so that confidence can be given to the relationships (or lack of) demonstrated by these studies. This would improve clarity in the field and eliminate ambiguity in interpretation of results. This systematic review further highlights the need for laboratory methodology standards to be developed for the use of the HFIM and has identified the key areas of inconsistency and we believe there is a need for a consensus standard checklist for minimum reporting. We have identified criteria we consider necessary for other researchers to be able to evaluate and reproduce an experiment, which we offer as a recommendation for reporting HFIM experiments (Table 5). We hope these recommendations will spark interest in setting a common standard to all HFIM studies investigating antimicrobial activity, similar to antimicrobial susceptibility guidelines (e.g. CLSI and EUCAST). These standards of reporting will also help reduce the barrier to entry for new laboratories setting up HFIM experiments. These recommendations can also pave the way for further conversations that the field could delve into. For instance, exploring thresholds for varying degrees of observed antimicrobial activity.

**Table 5.** Recommendations for suggested reporting of specifications in the HFIM experiments

Section	HFIM feature	Further explanation
Descriptive specifications	primary study aim	main research question of the HFIM study
	microbial species	microbial species inoculated into the hollow fibre cartridge
	antimicrobial(s)	antimicrobial(s) administered to the HFIM system
	duration	duration of the HFIM experiment in days
Technical specifications	mimicked dose^a^	dose being mimicked in the HFIM system in mg/L
	*C*_max_	peak concentration of antimicrobial(s) in mg/L
	*t*_½_	elimination half-life
	*T*_max_ ^a^	time taken to reach *C*_max_ in h
	*C*_min_ ^a^	lowest concentration of the drug in a dosing interval
	*Τ*	time between antimicrobial dose administrations
	cartridge source	manufacturer of the cartridge used and catalogue number
	fibre type	cartridge fibre type (e.g. cellulosic, polysulfone or polypropylene)
	flow rate	rate between the central compartment and the hollow fibre cartridge and rate from the diluent compartment
	pump model	pump models used
	tubing^a^	bore size and length of tubing used in the central compartment
	administration	route and details of administration, e.g. bolus or infusion, with details of rate and volume
Microbiological specifications	medium	name and manufacturer of medium used in the HIFM
	control	details of control experiment, e.g. drug-free arm
	contamination	measurement of sterility in the cartridge and the central compartment
	inoculum	method used to determine inoculum quantification stated
	cfu	cfu of the microbe quantified from the cartridge sampling
	viability^a^	cell viability markers beyond cfu (e.g. flow cytometry)
	resistance^a^	resistance of microbial sample phenotypically quantified
	genotyping^a^	molecular testing of the microbial sample
	biological repeat	number of repeat testing of single microbial species
	technical repeat	number of repeat testing of endpoint measures, e.g. cfu

a Additional parameters not required for minimal reporting.

We appreciate reporting of some parameters may be challenging and therefore propose some solutions.

For example, suitability of cartridge types (cellulosic, polysulfone etc.) for specific antimicrobials may not be available. For these situations, we suggest it is more important to check equilibration and binding for the chosen cartridge fibre type in these novel studies. We appreciate, with a wide variety of pump brands, that reporting the specific pump model and settings could get complex. We propose a standardized method of reporting flow rates (e.g. mL/min) that were used in the experiment that can be replicated by others. Factors that affect the time taken for equilibrium to be reached between the central reservoir and hollow fibre compartment should be reported, as often there can be a significant delay between input of the antimicrobial to the central reservoir and distribution to the cartridge. For example, the volume of the dose administered relative to the volume of the central compartment, or the flow rate of the dose infused relative to the volume of the central compartment, is also a critical parameter that affects the attainment of *C*_max_ at *T*_max_. Although tubing length from the drug medium and waste compartment is not important, the length and bore size of tubing between the central compartment and the cartridge has an impact on the distribution and equilibration rate of the drug.

As the HFIM can be used to understand the dynamic microbial response to antimicrobials, wherever possible we should take the opportunity to undertake further analyses of the microbial population sampled, for example genetic sequencing, transcriptomics or flow cytometry. We understand the cost of performing biological replicates for HFIM experiments is high, with several cartridges required per experiment. In instances where repurposed cartridges (e.g. dialysis fibres) are used instead of proprietary cartridges on cost grounds we strongly advise reporting this. In addition, cartridge pore size should also be reported; this can be captured by manufacturer and catalogue number. Further to this, we appreciate that for slow-growing organisms, such as mycobacteria, performing biological repeats under time constraints may be challenging. In these instances, we suggest performing static time–kill experiments in triplicate, to build a model hypothesis that can be simulated in the HFIM.

### Conclusions

This systematic review found wide variability in reporting, with most HFIM studies not providing sufficient information for their results to be evaluated. This creates difficulty in data comparison and reproducibility of studies. We believe there is scope for developing standards of reporting widely accepted as the recommendation for future HFIM studies.

## Funding

This systematic review was performed as part of Z.S.'s PhD studentship that was partially funded by an educational grant from Shionogi B.V. and by the University College London Institute for Global Health (IGH) and Centre for Clinical Microbiology (CCM). F.K. was supported by a United Kingdom Medical Research Council (MRC) Fellowship (Grant Number P014534) and a Sir Henry Dale Fellowship jointly funded by the Wellcome Trust and the Royal Society (Grant Number 220587/Z/20/Z).

## Transparency declarations

C.L. is an employee of Shionogi Europe and D.M. is an employee of QIAGEN and holds QIAGEN shares. All other authors: none to declare.

## Supplementary data

Tables S1 to S4 are available as Supplementary data at *JAC* Online.

## Supplementary Material

dkab160_Supplementary_Data
