# Supplementary material for: Application of the hollow fibre infection model (HFIM) in antimicrobial development: a systematic review and recommendations of reporting
Source: J Antimicrob Chemother. 2021 Jun 28;76(9):2252–9. doi: 10.1093/jac/dkab160 (PMC8361333; doi:10.1093/jac/dkab160)
Supplement: dkab160_Supplementary_Data [file dkab160_supplementary_data.docx]

**Supplementary data**

| Table S1. PICO framework | |
| --- | --- |
| *P - population* | Microbial cell culture in hollow fibre in vitro model |
| *I – intervention* | Antibiotic/combinations and doses  Experiment duration  Experiment repeats  Modulation of environmental conditions to reflect different physiological compartments such as blood, lung, CSF, tissue for example differences in pH, pO2/pCO2, albumin levels, free iron, etc.  Polymicrobial cultures/ multi-infection models  The use of cells for example THP-1 or macrophages for intracellular pathogens |
| *C - comparator* | n/a |
| *O - outcome* | CFUs  Viable/dead cell quantification  Inhibition of growth  Development of tolerance/resistance  Drug concentrations  Genotyping; acquired resistance  Multi infection interactions  Reporting of growth control results or comparison to best known comparator as a standard drug treatment regimen |

| Table S2. Search strategy for peer reviewed databases | | | |
| --- | --- | --- | --- |
| Database | **Step** | **Searching strategy** | **Number of articles** |
| EMBASE® | #1 | microorganism.mp. or microorganism or  bacterium.mp. or bacterium or bacteria.mp. or  parasite or parasite.mp. or  mycobacterium.mp. or mycobacterium or | 1172685 |
|  | #2 | antibiotic agent.mp. or antibiotic agent or  antibiotic.mp. or antiinfective agent.mp. or  antiinfective agent or antimicrobial.mp. | 930463 |
|  | #3 | hollow fibre infection model.mp. or hollow fiber infection model.mp. or hollow fibre bioreactor.mp. or hollow fiber bioreactor.mp. or hollow fibre reactor.mp. or hollow fiber reactor/ or HFIM.mp. or hollow fibre.mp. or hollow fiber.mp. | 8250 |
|  | #4 | #1 AND #2 AND #3 AND #4 AND Language: English | 127 |
| MEDLINE® | #1 | microorganism.mp. or microorganism or bacterium.mp. or bacterium or parasite or parasite.mp. or mycobacterium.mp. or Mycobacterium/ orbacteria.mp. | 827145 |
|  | #2 | antibiotic agent.mp. or antibiotic agent or antibiotic.mp. or antiinfective agent.mp. or antiinfective agent or antimicrobial.mp. | 374114 |
|  | #3 | hollow fibre infection model.mp. or hollow fiber infection model.mp. or hollow fibre bioreactor.mp. or hollow fiber bioreactor.mp. or hollow fibre reactor.mp. or hollow fiber reactor/ or HFIM.mp. or hollow fibre.mp. or hollow fiber.mp. | 4730 |
|  | #4 | #1 AND #2 AND #3 AND #4 AND English language | 40 |
| BIOSIS® | #1 | TS=(bacteria) OR TS=(bacterium) OR TS=(microbes) OR TS=(microorganism) OR TS=(parasite) OR TS=(parasites) OR  TS=(mycobacteria) OR TS=(mycobacterium) Indexes=BCI Timespan=1926-2020 | 5308588 |
|  | #2 | TS= (hollow fibre infection model) OR TS= (hollow fiber infection model) OR TS=(hollow fibre reactor) OR TS=(hollow fiber reactor) OR TS=(hollow fibre bioreactor) OR TS=(hollow fiber bioreactor) OR TI=(HFIM) | 1626 |
|  | #3 | TS=(antibiotic) OR TS=(antibiotics) OR TS=(antinfective) OR TS=(antimicrobial) OR TS=(antimicrobials) | 458042 |
|  | #4 | #1 AND #2 AND #3 AND English language | 93 |
| PubMed® | #1 | microorganism z[MeSH Terms] OR microbiology[MeSH Subheading] OR bacteria OR parasite | 2,705,215 |
|  | #2 | Pharmacokinetics[MeSH Subheading] OR Drug effects[MeSH Subheading] OR antibiotic OR antimicrobial OR antiinfective | 4,530,012 |
|  | #3 | (hollow fibre infection model) OR (hollow fiber infection model) OR (hollow fiber infection) OR (hollow fibre infection) OR (hollow fiber reactor) OR (hollow fibre reactor) OR (hollow fiber bioreactor) OR (hollow fibre bioreactor) | 1,381 |
|  | #4 | #1 AND #2 AND #3 AND Language: English | 95 |
| Scopus® | #1 | TITLE-ABS-KEY ("microorganism") OR ("bacteria") OR ("parasite") OR ("mycobacteria") | 3,087,070 |
|  | #2 | TITLE-ABS-KEY ( "antibiotic" ) OR ( ‎‎ "antimicrobial" ) OR ( "antiinfective") | 1,958,402 |
|  | #3 | TITLE-ABS-KEY ( "hollow fibre infection model" ) OR ( "hollow fiber infection model" ) OR ( "hollow fibre bioreactor" ) OR ( "hollow fiber bioreactor" ) OR ( "HFIM") | 3240 |
|  | #4 | #1 AND #2 AND #3 LIMIT-TO ( LANGUAGE , "English" ) LIMIT-TO ( DOCTYPE , "ar" ) | 108 |
| Cochrane® | #1 | (‎( "antibiotic" ) OR ( ‎‎ "antimicrobial" ) OR ( "antiinfective")‎):ti,ab,kw | 40513 |
|  | #2 | ‎("microorganism") OR ("bacteria") OR ("parasite") OR ‎‎("mycobacteria")‎ | 17223 |
|  | #3 | ‎( "hollow fibre infection model" ) OR ( "hollow fiber ‎infection model" ) OR ( "hollow fibre bioreactor" ) OR ( ‎‎"hollow fiber bioreactor" ) OR ( "HFIM")‎ | 1 |
|  | #4 | #1 AND #2 AND #3 | 0 |

| **Table S3.** Definition of information extracted from included publications | |
| --- | --- |
| **Parameter** | **Defined as** |
| Main study aim | Primary aim of the HFIM study |
| Microbe investigated | Species investigated in the HFIM |
| Antimicrobial investigated | Antimicrobial(s) investigated in the HFIM |
| Antimicrobial administration | Route of administration of antimicrobial to HFIM e.g. infusion or bolus |
| Duration | Length of HFIM experiment, represented in days |
| Cartridge | Cartridge manufacturer |
| Fibre type | The type of fibre in the cartridge e.g. cellulosic/polysulfone |
| Pump dynamics | The flow rate and time settings or rpm and pump model |
| PK parameters | Parameters that define the pharmacokinetic |
| Dose administration | Dose mimicked and administration route e.g., bolus or infusion |
| pH | Measurement of the activity of hydrogen ions in solution |
| Tubing bore size | Internal diameter of the tubing |
| Tubing filtration | Filtration suitability of tubing for studied antibiotic |
| Tubing length | Length of the tubing used to connect the systems reservoirs |
| Media | Liquid broth pumped through the hollow fibre system |
| Contamination | Measurement of sterility in the systems reservoirs |
| Control | Experiment with no independent variable e.g. drug free experiment |
| Inoculum method | Method used to determine inoculum quantification stated |
| CFUs | CFUs of the studied microbe quantified from the cartridge sampling |
| Viability | Other cell viability markers beyond CFU measured |
| Resistance | Resistance of cartridge sample phenotypically quantified |
| Genotyping ‎ | Molecular testing of the cartridge sample |
| Biological repeat | Repeat testing of microbial species |
| Technical repeat | Repeat testing of endpoint measures, e.g. CFUs |

**Table S4.** Descriptive characteristics of all 129 studies included in the systematic review including microbial species and antibiotic/combinations investigated in the HFIMs

| Pubmed ID | Publication title | Publication primary aim | Species investigated | Antibiotic/combination investigated |
| --- | --- | --- | --- | --- |
| 25182633 | Evaluation of meropenem regimens suppressing emergence of resistance in acinetobacter baumannii with human simulated exposure in an in vitro intravenous-infusion hollow-fiber infection model | antimicrobial resistance | A. baumannii | Meropenem |
| 27067330 | Paradoxical effect of polymyxin B: High drug exposure amplifies resistance in Acinetobacter baumannii | antimicrobial resistance | A. baumannii | Polymyxin B |
| 18725438 | Pharmacodynamic Modeling of Aminoglycosides against Pseudomonas aeruginosa and Acinetobacter baumannii: Identifying Dosing Regimens To Suppress Resistance Development | antimicrobial resistance | A. baumannii and P. aeruginos | Multiple: Amikacin or Gentamicin |
| 24041894 | Hollow-Fiber Pharmacodynamic Studies and Mathematical Modeling To Predict the Efficacy of Amoxicillin for Anthrax Postexposure Prophylaxis in Pregnant Women and Children | antimicrobial resistance | B. anthracis | Amoxicillin |
| 19687233 | Impact of spore biology on the rate of kill and suppression of resistance in Bacillus anthracis. | antimicrobial resistance | B. anthracis | Moxifloxacin |
| 23774429 | Relationship between ceftolozane-tazobactam exposure and drug resistance amplification in a hollow-fiber infection model | antimicrobial resistance | E. coli | Combination: Ceftolozane/Tazobactam |
| 26124169 | Pharmacodynamics of fosfomycin: Insights into clinical use for antimicrobial resistance | antimicrobial resistance | E. coli | Combination: Fosfomycin and Meropenem |
| 27270274 | Relationship between fosfomycin exposure and amplification of Escherichia coli subpopulations with reduced susceptibility in a hollow-fiber infection model | antimicrobial resistance | E. coli | Fosfomycin |
| 22232279 | Temporal interplay between efflux pumps and target mutations in development of antibiotic resistance in Escherichia coli | antimicrobial resistance | E. coli | Levofloxacin |
| 28505268 | Pharmacodynamics of dose-escalated 'front-loading' polymyxin B regimens against polymyxin-resistant mcr-1-harbouring Escherichia coli. | antimicrobial resistance | E. coli | Polymyxin B |
| 20498316 | In vitro system for modeling influenza A virus resistance under drug pressure | antimicrobial resistance | Influenza A | Amantadine |
| 28158470 | Determining beta-lactam exposure threshold to suppress resistance development in Gram-negative bacteria | antimicrobial resistance | K. pneumoniae and P. aeruginosa | Combination: Cefepime and Ceftazidime and Meropenem |
| 17116679 | The relationship between quinolone exposures and resistance amplification is characterized by an inverted U: A new paradigm for optimizing pharmacodynamics to counterselect resistance | antimicrobial resistance | K. pneumoniae and S. aureus | Garenoxacin |
| 22751536 | The antibiotic resistance arrow of time: Efflux pump induction is a general first step in the evolution of mycobacterial drug resistance | antimicrobial resistance | M. avium | Azithromycin |
| 21896907 | Pharmacokinetic mismatch does not lead to emergence of isoniazid or rifampin-resistant Mycobacterium tuberculosis but to better antimicrobial effect: A new paradigm for antituberculosis drug scheduling | antimicrobial resistance | M. tuberculosis | Combination: Isoniazid and Rifampin |
| 28584143 | Linezolid dose that maximizes sterilizing effect while minimizing toxicity and resistance emergence for tuberculosis | antimicrobial resistance | M. tuberculosis | Linezolid |
| 30249693 | Activity of Moxifloxacin against Mycobacterium tuberculosis in Acid Phase and Nonreplicative-Persister Phenotype Phase in a Hollow-Fiber Infection Model. | antimicrobial resistance | M. tuberculosis | Moxifloxacin |
| 25313208 | Impact on Resistance of the Use of Therapeutically Equivalent Generics: the Case of Ciprofloxacin | antimicrobial resistance | P. aeruginosa | Ciprofloxacin |
| 22005996 | Resistance emergence mechanism and mechanism of resistance suppression by tobramycin for cefepime for Pseudomonas aeruginosa | antimicrobial resistance | P. aeruginosa | Combination: Cefepime and Tobramycin |
| 25070105 | Relationship between ceftolozane-tazobactam exposure and selection for Pseudomonas aeruginosa resistance in a hollow-fiber infection model | antimicrobial resistance | P. aeruginosa | Combination: Ceftolozane/Tazobactam |
| 20308371 | Impact of different carbapenems and regimens of administration on resistance emergence for three isogenic Pseudomonas aeruginosa strains with differing mechanisms of resistance | antimicrobial resistance | P. aeruginosa | Combination: Doripenem and Imipenem |
| 16304153 | Optimization of meropenem minimum concentration/MIC ratio to suppress in vitro resistance of Pseudomonas aeruginosa | antimicrobial resistance | P. aeruginosa | Combination: Meropenem and Tobramycin |
| 24002098 | Impact of bolus dosing versus continuous infusion of piperacillin and tazobactam on the development of antimicrobial resistance in Pseudomonas aeruginosa | antimicrobial resistance | P. aeruginosa | Combination: Piperacillin/tazobactam |
| 17431788 | Modeling of microbial population responses to time-periodic concentrations of antimicrobial agents | antimicrobial resistance | P. aeruginosa | Levofloxacin |
| 17913722 | Mathematical modelling response of Pseudomonas aeruginosa to meropenem | antimicrobial resistance | P. aeruginosa | Meropenem |
| 19738009 | In vitro activity of ceftaroline against methicillin-resistant Staphylococcus aureus and heterogeneous vancomycin-intermediate S. aureus in a hollow fiber model | antimicrobial resistance | S. aureus | Ceftaroline |
| 29091195 | Ceftaroline efficacy against high-MIC clinical Staphylococcus aureus isolates in an in vitro hollow-fibre infection model | antimicrobial resistance | S. aureus | Ceftaroline |
| 21343454 | Mutant prevention concentration-based pharmacokinetic/pharmacodynamic indices as dosing targets for suppressing the enrichment of levofloxacin- resistant subpopulations of Staphylococcus aureus | antimicrobial resistance | S. aureus | Levofloxacin |
| 22526313 | Front-loaded linezolid regimens result in increased killing and suppression of the accessory gene regulator system of Staphylococcus aureus | antimicrobial resistance | S. aureus | Linezolid |
| 23545533 | Evaluation of Ceftaroline Activity against Heteroresistant Vancomycin-Intermediate Staphylococcus aureus and Vancomycin-Intermediate Methicillin-Resistant S-aureus Strains in an In Vitro Pharmacokinetic/Pharmacodynamic Model: Exploring the Seesaw Effect"" | antimicrobial resistance | S. aureus | Multiple: Ceftaroline or Vancomycin |
| 28962026 | Pharmacodynamics of teicoplanin against MRSA | antimicrobial resistance | S. aureus | Teicoplanin |
| 26755499 | Pharmacodynamics of vancomycin for CoNS infection: experimental basis for optimal use of vancomycin in neonates | antimicrobial resistance | S. epidermidis and S. capitis | Vancomycin |
| 21486959 | Comparative Efficacies of Candidate Antibiotics against Yersinia pestis in an In Vitro Pharmacodynamic Model | antimicrobial resistance | Y. pestis | Multiple: Ampicilllin or Ciprofloxacin or Doxycycline or Gentamicin or Meropenem or Moxifloxacin or Streptomycin |
| 20134256 | Induced death of Escherichia coli encapsulated in a hollow fiber membrane as observed in vitro or after subcutaneous implantation | Determine conditions for drug production | E. coli | Tetracycline |
| 18553771 | DUAL AEROBIC HOLLOW-FIBER BIOREACTOR FOR CULTIVATION OF STREPTOMYCES-AUREOFACIENS | Determine conditions for drug production | Streptomycetaceae | Tetracycline |
| 21393141 | Modelling biphasic killing of fluoroquinolones: guiding optimal dosing regimen design | dose finding | E.coli and S. aureus | Levofloxacin |
| 27742638 | Linezolid for Infants and Toddlers With Disseminated Tuberculosis: First Steps | dose finding | M. tuberculosis | Linezolid |
| 18505848 | Quantitative assessment of combination antimicrobial therapy against multidrug-resistant Acinetobacter baumannii | drug combinations | A. baumannii | Combination: Amikacin and Cefepime and Levofloxacin |
| 28052852 | High-Dose Ampicillin-Sulbactam Combinations Combat Polymyxin-Resistant Acinetobacter baumannii in a Hollow-Fiber Infection Model. | drug combinations | A. baumannii | Combination: Ampicillin/Sulbactam and Meropenem and Polymixin B |
| 28333347 | Polymyxin-resistant, carbapenem-resistant Acinetobacter baumannii is eradicated by a triple combination of agents that lack individual activity | drug combinations | A. baumannii | Combination: Ampicillin/Sulbactam and Meropenem and Polymixin B |
| 29339388 | Combating Carbapenem-Resistant Acinetobacter baumannii by an Optimized Imipenem-plus-Tobramycin Dosage Regimen: Prospective Validation via Hollow-Fiber Infection and Mathematical Modeling. | drug combinations | A. baumannii | Combination: Imipenem and Tobramycin |
| 27324776 | In vitro activity of polymyxin B in combination with various antibiotics against extensively drug-resistant Enterobacter cloacae with decreased susceptibility to polymyxin B | drug combinations | E. cloacae | Combination: Polymyxin B and Tigecycline |
| 28743810 | Polymyxin combinations combat Escherichia coli harboring mcr-1 and blaNDM-5: Preparation for a postantibiotic Era | drug combinations | E. coli | Combination: Amikacin and Aztreonam and Polymixin B |
| 27931793 | In vitro pharmacodynamic evaluation of ceftolozane/tazobactam against beta-lactamase-producing Escherichia coli in a hollow-fibre infection model | drug combinations | E. coli | Combination: Ceftolozane/Tazobactam |
| 27795375 | Evaluating polymyxin B-based combinations against carbapenemresistant Escherichia coli in time-kill studies and in a hollow-fiber infection model | drug combinations | E. coli | Combination: Polymyxin B and Tigecycline |
| 28145085 | Prediction of in vivo and in vitro infection model results using a semimechanistic model of avibactam and aztreonam combination against multidrug resistant organisms | drug combinations | K. pneumoniae | Combination: Aztreonam and Avibactam |
| 22024819 | Pharmacodynamics of beta-lactamase inhibition by NXL104 in combination with ceftaroline: Examining organisms with multiple types of beta-lactamases | drug combinations | K. pneumoniae | Combination: Ceftaroline and NXL104 |
| 28444224 | Pharmacodynamics of colistin and fosfomycin: A 'treasure trove' combination combats KPC-producing Klebsiella pneumoniae | drug combinations | K. pneumoniae | Combination: Colistin and Fosfomycin |
| 29486233 | Polymyxin B and fosfomycin thwart KPC-producing Klebsiella pneumoniae in the hollow-fibre infection model | drug combinations | K. pneumoniae | Combination: Fosfomycin and Polymyxin B |
| 22330927 | Novel Modeling Framework To Guide Design of Optimal Dosing Strategies for beta-Lactamase Inhibitors | drug combinations | K. pneumoniae | Combination: Imipenem and Relabactam |
| 28167549 | New Polymyxin B Dosing Strategies To Fortify Old Allies in the War against KPC-2-Producing Klebsiella pneumoniae. | drug combinations | K. pneumoniae | Combination: Meropenem and Polymyxin B and Rifampin |
| 25691628 | In vitro pharmacodynamics of various antibiotics in combination against extensively drug-resistant Klebsiella pneumoniae | drug combinations | K. pneumoniae | Combination: Meropenem and Tigecycline |
| 24687507 | Activities of ceftazidime and avibactam against beta-lactamase-producing Enterobacteriaceae in a hollow-fiber pharmacodynamic model | drug combinations | K. pneumoniae and E. cloacae and C. freundi | Combination: Ceftazidime/Avibactam |
| 27795380 | Pharmacodynamics of Aerosolized Fosfomycin and Amikacin against Resistant Clinical Isolates of Pseudomonas aeruginosa and Klebsiella pneumoniae in a Hollow-Fiber Infection Model: Experimental Basis for Combination Therapy. | drug combinations | K. pneumoniae and P. aeruginosa | Combination: Amikacin and Fosfomycin |
| 27458221 | Failure of the amikacin, cefoxitin, and clarithromycin combination regimen for treating pulmonary Mycobacterium abscessus infection | drug combinations | M. abscessus | Combination: Amikacin and Cefozitin and Clarithromycin |
| 28922805 | Failure of the azithromycin and ethambutol combination regimen in the hollow-fibre system model of pulmonary Mycobacterium avium infection is due to acquired resistance | drug combinations | M. avium | Combination: Azithromycin and Ethambutol |
| 28922808 | The discovery of ceftazidime/avibactam as an anti-Mycobacterium avium agent | drug combinations | M. avium | Combination: Ceftazidime/Avibactam |
| 28922809 | A novel ceftazidime/avibactam, rifabutin, tedizolid and moxifloxacin (CARTM) regimen for pulmonary Mycobacterium avium disease | drug combinations | M. avium | Combination: Ceftazidime/Avibactam and Tedizolid |
| 28922810 | A 'shock and awe' thioridazine and moxifloxacin combination-based regimen for pulmonary Mycobacterium avium-intracellulare complex disease. | drug combinations | M. avium | Combination: Moxifloxacin and Thioridazine |
| 28875168 | Ceftazidime-avibactam has potent sterilizing activity against highly drug-resistant tuberculosis. | drug combinations | M. tuberculosis | Combination: Ceftazidime/Avibactam |
| 27742640 | A Faropenem, Linezolid, and Moxifloxacin Regimen for Both Drug-Susceptible and Multidrug-Resistant Tuberculosis in Children: FLAME Path on the Milky Way | drug combinations | M. tuberculosis | Combination: Isoniazid and Faropenem and Linezolid and Moxifloxacin and Pyrazinamide and Rifampin |
| 27742639 | Concentration-Dependent Synergy and Antagonism of Linezolid and Moxifloxacin in the Treatment of Childhood Tuberculosis: The Dynamic Duo | drug combinations | M. tuberculosis | Combination: Linezolid and Moxifloxacin |
| 30496456 | Multiparameter Responses to Tedizolid Monotherapy and Moxifloxacin Combination Therapy Models of Children With Intracellular Tuberculosis | drug combinations | M. tuberculosis | Combination: Linezolid and Moxifloxacin and Tedizolid |
| 25003557 | Analysis of combination drug therapy to develop regimens with shortened duration of treatment for tuberculosis. | drug combinations | M. tuberculosis | Combination: Linezolid and Rifampicin |
| 30496465 | Efficacy Versus Hepatotoxicity of High-dose Rifampin, Pyrazinamide, and Moxifloxacin to Shorten Tuberculosis Therapy Duration: There Is Still Fight in the Old Warriors Yet! | drug combinations | M. tuberculosis | Combination: Moxifloxacin and Pyrazinamide and Rifampin |
| 20802826 | The combination of rifampin plus moxifloxacin is synergistic for suppression of resistance but antagonistic for cell kill of Mycobacterium tuberculosis as determined in a hollow-fiber infection model | drug combinations | M. tuberculosis | Combination: Moxifloxacin and Rifampin |
| 29530842 | Evaluation of ceftolozane-tazobactam in combination with meropenem against pseudomonas aeruginosa sequence type 175 in a hollow-fiber infection model | drug combinations | P. aeruginosa | Combination: Ceftolozane/Tazobactam and Meropenem |
| 30104278 | Meropenem Combined with Ciprofloxacin Combats Hypermutable Pseudomonas aeruginosa from Respiratory Infections of Cystic Fibrosis Patients. | drug combinations | P. aeruginosa | Combination: Ciprofloxacin and Meropenem |
| 25712313 | Colistin and doripenem combinations against Pseudomonas aeruginosa: profiling the time course of synergistic killing and prevention of resistance. | drug combinations | P. aeruginosa | Combination: Colistin and Doripenem |
| 29632010 | Polymyxin b in combination with enrofloxacin exerts synergistic killing against extensively drug-resistant pseudomonas aeruginosa | drug combinations | P. aeruginosa | Combination: Enrfloxacin and Polymixin B |
| 30249700 | The Combination of Fosfomycin plus Meropenem Is Synergistic for Pseudomonas aeruginosa PAO1 in a Hollow-Fiber Infection Model. | drug combinations | P. aeruginosa | Combination: Fosfomycin and Meropenem |
| 20368395 | The Combination of Meropenem and Levofloxacin Is Synergistic with Respect to both Pseudomonas aeruginosa Kill Rate and Resistance Suppression | drug combinations | P. aeruginosa | Combination: Levofloxacin and Meropenem |
| 29437610 | Optimization of a Meropenem-Tobramycin Combination Dosage Regimen against Hypermutable and Nonhypermutable Pseudomonas aeruginosa via Mechanism-Based Modeling and the Hollow-Fiber Infection Model. | drug combinations | P. aeruginosa | Combination: Meropenem and Tobramycin |
| 31636062 | Meropenem-Tobramycin Combination Regimens Combat Carbapenem-Resistant Pseudomonas aeruginosa in the Hollow-Fiber Infection Model Simulating Augmented Renal Clearance in Critically III Patients | drug combinations | P. aeruginosa | Combination: Meropenem and Tobramycin |
| 29463528 | Optimization and Evaluation of Piperacillin-Tobramycin Combination Dosage Regimens against Pseudomonas aeruginosa for Patients with Altered Pharmacokinetics via the Hollow-Fiber Infection Model and Mechanism-Based Modeling. | drug combinations | P. aeruginosa | Combination: Piperacillin and Tobramycin |
| 29636741 | Differential Activity of the Combination of Vancomycin and Amikacin on Planktonic vs. Biofilm-Growing Staphylococcus aureus Bacteria in a Hollow Fiber Infection Model | drug combinations | S. aureus | Combination: Amikacin and Vancomycin |
| 27494922 | Polymyxin B in combination with doripenem against heteroresistant Acinetobacter baumannii: Pharmacodynamics of new dosing strategies | drug development | A. baumannii | Combination: Doripenem and Polymixin B |
| 18725437 | Is 60 days of ciprofloxacin administration necessary for postexposure prophylaxis for Bacillus anthracis?. | drug development | B. anthracis | Ciprofloxacin |
| 22155821 | Impact of spores on the comparative efficacies of five antibiotics for treatment of Bacillus anthracis in an in vitro hollow fiber pharmacodynamic model | drug development | B. anthracis | Combination: Ciprofloxacin and Doxycycline and Linezolid and Moxifloxacin and Meropenem |
| 29311092 | Repurposing and Reformulation of the Antiparasitic Agent Flubendazole for Treatment of Cryptococcal Meningoencephalitis, a Neglected Fungal Disease | drug development | Cryptococcus neoformans | Flubendazole |
| 19589810 | Pharmacodynamics of moxifloxacin against a high inoculum of Escherichia coli in an in vitro infection model | drug development | E. coli | Moxifloxacin |
| 31109982 | Pharmacodynamics of Tebipenem: New Options for Oral Treatment of Multidrug-Resistant Gram-Negative Infections. | drug development | E. coli | Tebipenem |
| 30397063 | Pharmacokinetics/Pharmacodynamics of Vaborbactam, a Novel Beta-Lactamase Inhibitor, in Combination with Meropenem | drug development | E. coli and K. pneumoniae and E. cloacae | Combination: Meropenem and Vaborbactam |
| 31611348 | Pharmacodynamics of ClpP-activating antibiotic combinations against Gram-positive pathogens | drug development | E. faecalis and S. aureus | Combination: ADEP4 and Ampicillin and Linezolid and Oxacillin |
| 29507068 | Exploring the Pharmacokinetic/Pharmacodynamic Relationship of Relebactam (MK-7655) in Combination with Imipenem in a Hollow-Fiber Infection Model | drug development | E.coli and K. pneumoniae and P. aeruginosa and S.marcescens | Combination: Imipenem and Relebactam |
| 8585738 | Efficacy of constant infusion of A-77003, an inhibitor of the human immunodeficiency virus type 1 (HIV-1) protease, in limiting acute HIV-1 infection in vitro | drug development | HIV | A-77003 |
| 11237841 | Hollow-fiber unit evaluation of a new human immunodeficiency virus type 1 protease inhibitor, BMS-232632, for determination of the linked pharmacodynamic variable | drug development | HIV | BMS-232633 |
| 22526311 | In Vitro Activity of MK-7655, a Novel beta-Lactamase Inhibitor, in Combination with Imipenem against Carbapenem-Resistant Gram-Negative Bacteria | drug development | K. pneumoniae and P. aeruginosa | Combination: Imipenem and MK-7655 |
| 27067317 | Moxifloxacin's Limited Efficacy in the Hollow-Fiber Model of Mycobacterium abscessus Disease | drug development | M. abscessus | Moxifloxacin |
| 31039251 | Minocycline treatment for pulmonary Mycobacterium avium complex disease based on pharmacokinetics/pharmacodynamics and Bayesian framework mathematical models | drug development | M. avium | Minocycline |
| 27458215 | Amikacin Optimal Exposure Targets in the Hollow-Fiber System Model of Tuberculosis | drug development | M. tuberculosis | Amikacin |
| 30496460 | d-Cycloserine Pharmacokinetics/Pharmacodynamics, Susceptibility, and Dosing Implications in Multidrug-resistant Tuberculosis: A Faustian Deal | drug development | M. tuberculosis | D-cycloserine |
| 30496457 | Ethionamide Pharmacokinetics/Pharmacodynamics-derived Dose, the Role of MICs in Clinical Outcome, and the Resistance Arrow of Time in Multidrug-resistant Tuberculosis | drug development | M. tuberculosis | Ethionamide |
| 30496459 | Gatifloxacin Pharmacokinetics/Pharmacodynamics-based Optimal Dosing for Pulmonary and Meningeal Multidrug-resistant Tuberculosis. | drug development | M. tuberculosis | Gatifloxacin |
| 30496461 | Levofloxacin Pharmacokinetics/Pharmacodynamics, Dosing, Susceptibility Breakpoints, and Artificial Intelligence in the Treatment of Multidrug-resistant Tuberculosis | drug development | M. tuberculosis | Levofloxacin |
| 26530386 | Preclinical Evaluations To Identify Optimal Linezolid Regimens for Tuberculosis Therapy | drug development | M. tuberculosis | Linezolid |
| 30597040 | Minocycline Immunomodulates via Sonic Hedgehog Signaling and Apoptosis and Has Direct Potency Against Drug-Resistant Tuberculosis. | drug development | M. tuberculosis | Minocycline |
| 30385322 | Dose optimization of moxifloxacin and linezolid against tuberculosis using mathematical modeling and simulation | drug development | M. tuberculosis | Multiple: Linezolid or Moxifloaxacin |
| 29581114 | Determination of the Dynamically Linked Indices of Fosfomycin for Pseudomonas aeruginosa in the Hollow Fiber Infection Model | drug development | P. aeruginosa | Fosfomycin |
| 16127031 | Pharmacodynamics of polymyxin B against Pseudomonas aeruginosa | drug development | P. aeruginosa | Polymyxin B |
| 8239581 | Dose ranging and fractionation of intravenous ciprofloxacin against Pseudomonas aeruginosa and Staphylococcus aureus in an in-vitro model of infection | drug development | P. aeruginosa and S. aureus | Ciprofloxacin |
| 23229481 | In vitro pharmacodynamics of AZD5206 against Staphylococcus aureus | drug development | S. aureus | AZD5206 |
| 19049435 | Impact of short-course quinolone therapy on susceptible and resistant populations of Staphylococcus aureus | drug development | S. aureus | Garenoxacin |
| 22083484 | Evaluation of Once-Daily Vancomycin against Methicillin-Resistant Staphylococcus aureus in a Hollow-Fiber Infection Model | drug development | S. aureus | Vancomycin |
| 11249827 | Pharmacodynamics of moxifloxacin and levofloxacin against Staphylococcus aureus and Staphylococcus epidermidis in an in vitro pharmacodynamic model | drug development | S. aureus and S. epidermidis | Multiple: Levofloxacin or Moxifloxacin |
| 27216055 | Thioridazine as chemotherapy for Mycobacterium avium complex diseases | intracellular pathogens | M. avium | Thioridazine |
| 29844047 | Clofazimine for the treatment of mycobacterium kansasii | intracellular pathogens | M. kansasii | Clofazimine |
| 25645830 | Rapid drug tolerance and dramatic sterilizing effect of moxifloxacin monotherapy in a novel hollow-fiber model of intracellular Mycobacterium kansasii disease. | intracellular pathogens | M. kansasii | Moxifloxacin |
| 29866864 | Linezolid kills acid-phase and nonreplicative-persister-phase mycobacterium tuberculosis in a hollow-fiber infection model | intracellular pathogens | M. tuberculosis | Linezolid |
| 27821440 | Bacterial Replication Rate Modulation in Combination with Antimicrobial Therapy: Turning the Microbe against Itself | Investigated modulation of bacterial replication rate | S. aureus | Levofloxacin |
| 22064533 | Simulated Antibiotic Exposures in an In Vitro Hollow-Fiber Infection Model Influence Toxin Gene Expression and Production in Community-Associated Methicillin-Resistant Staphylococcus aureus Strain MW2 | Investigating antibiotic effects on toxic gene exprression | S. aureus | Multiple: Clindamycin or Linezolid or Minocycline or Trimethoprim-sulfamethoxazole or Vancomycin |
| 26643339 | Amikacin Pharmacokinetics/Pharmacodynamics in a Novel Hollow-Fiber Mycobacterium abscessus Disease Model | modelling IV timecourse | M. abscessus | Amikacin |
| 26926649 | Tigecycline Is Highly Efficacious against Mycobacterium abscessus Pulmonary Disease. | modelling IV timecourse | M. abscessus | Tigecycline |
| 20231389 | Ethambutol optimal clinical dose and susceptibility breakpoint identification by use of a novel pharmacokinetic-pharmacodynamic model of disseminated intracellular Mycobacterium avium. | modelling IV timecourse | M. avium | Ethambutol |
| 27231278 | Effect of different renal function on antibacterial effects of piperacillin against Pseudomonas aeruginosa evaluated via the hollow-fibre infection model and mechanism-based modelling | modelling IV timecourse | P. aeruginosa | Combination: Piperacillin/tazobactam |
| 26711763 | Sequential Evolution of Vancomycin-Intermediate Resistance Alters Virulence in Staphylococcus aureus: Pharmacokinetic/Pharmacodynamic Targets for Vancomycin Exposure | modelling IV timecourse | S. aureus | Vancomycin |
| 23180085 | Optimizing hollow-fiber-based pharmacokinetic assay via chemical stability study to account for inaccurate simulated drug clearance of rifampicin | pharmacodynamic interactions | A. baumannii | Combination: Polymyxin B and Rifampicin |
| 16304178 | Effective antimicrobial regimens for use in humans for therapy of Bacillus anthracis infections and postexposure prophylaxis | pharmacodynamic interactions | B. anthracis | Levofloxacin |
| 29180527 | Azithromycin pharmacodynamics against persistent haemophilus influenzae in chronic obstructive pulmonary disease | pharmacodynamic interactions | Haemophilus | Azithromycin |
| 25870053 | Pharmacokinetic Determinants of Virological Response to Raltegravir in the In Vitro Pharmacodynamic Hollow-Fiber Infection Model System | pharmacodynamic interactions | HIV | Raltegravir |
| 28922806 | Linezolid as treatment for pulmonary Mycobacterium avium disease | pharmacodynamic interactions | M. avium | Linezolid |
| 28922807 | Tedizolid is highly bactericidal in the treatment of pulmonary Mycobacterium avium complex disease | pharmacodynamic interactions | M. avium | Tedizolid |
| 24041886 | Thioridazine pharmacokinetic-pharmacodynamic parameters Wobble" during treatment of tuberculosis: a theoretical basis for shorter-duration curative monotherapy with congeners" | pharmacodynamic interactions | M. tuberculosis | Combination: Isoniazid and Pyrazinamide and Rifampicin and Thioridazine |
| 28264846 | Substantial Impact of Altered Pharmacokinetics in Critically Ill Patients on the Antibacterial Effects of Meropenem Evaluated via the Dynamic Hollow-Fiber Infection Model. | pharmacodynamic interactions | P. aeruginosa | Meropenem |
| 16870751 | Comparative pharmacodynamics of gentamicin against Staphylococcus aureus and Pseudomonas aeruginosa | pharmacodynamic interactions | P. aeruginosa and S. aureus | Gentamicin |
| 29461629 | Development and validation of a LC-MS/MS method for quantitation of fosfomycin - Application to in vitro antimicrobial resistance study using hollow-fiber infection model | PK assay development | E. coli | Fosfomycin |
| 30652018 | Combining LC-MS/MS and hollow-fiber infection model for real-time quantitation of ampicillin to antimicrobial resistance. | Proof of concept for real-time quantification of ampicillin | E. coli | Ampicillin |
| 31504558 | Can phenotypic data complement our understanding of antimycobacterial effects for drug combinations? | drug combinations | M. komossense | Combination: ‎Isoniazid and Rifampicin |
| 31838036 | Effect of drug combinations on the kinetics of antibiotic resistance emergence in Escherichia coli CFT073 using an in vitro hollow-fibre infection model | antimicrobial resistance | E. coli | Combination: ‎Ampicillin, Ciprofloxacin and Fosfomycin |
